# Influence of Foraging Effort and Diet on Maternal Energy Stores and Offspring Weaning Mass in Gray Seals (*Halichoerus grypus*)

**DOI:** 10.1002/ece3.74536

**Published:** 2026-10-28

**Authors:** M. Henry‐Adams, W. D. Bowen, C. E. den Heyer, S. L. C. Lang, S. J. Iverson

**Affiliations:** ^1^ Department of Biology, Life Science Centre Dalhousie University Halifax Nova Scotia Canada; ^2^ Bedford Institute of Oceanography Fisheries and Oceans Canada Dartmouth Nova Scotia Canada; ^3^ Northwest Atlantic Fisheries Centre Fisheries and Oceans Canada St. John's Newfoundland and Labrador Canada

**Keywords:** foraging effort, gray seal, hidden Markov model, individual variation, quantitative fatty acid signature analysis

## Abstract

Understanding how foraging behavior and diet influence individual reproductive success is a key interest in evolutionary ecology. This is particularly true for capital breeding species where the accumulation and storage of energy to support offspring is critical, often following the “bigger‐is‐better” hypothesis. Using data collected from female gray seals ( *Halichoerus grypus* ) at Sable Island, Canada, between 1995 and 2018, we tested the hypothesis that differences in foraging effort and diet prior to the breeding season, estimated through satellite telemetry, stable isotope (SI) signatures, and quantitative fatty acid signature analysis (QFASA), respectively, affect reproductive success. There was no relationship between dominant prey type, the energy density of estimated diets, and maternal postpartum mass (MPPM). Females consuming a diet of lower diversity and with more positive δ^13^C estimates, possibly indicating more benthic foraging, had higher MPPM. MPPM was the best predictor of offspring mass at weaning. However, metrics of maternal foraging effort and diet alone did not influence pup weaning mass when MPPM was included in the model. These results suggest that different foraging strategies and diets can lead to sufficient body energy storage that enables reproductive success in a capital breeder. Considering this colony was increasing rapidly during the study period, the influence of foraging effort and diet might differ in a stable or declining population where intraspecific competition may be more evident.

## Graphical abstract

Understanding how foraging behavior and diet influences individual reproductive success is a key interest in evolutionary ecology. Using data collected from female gray seals ( *Halichoerus grypus* ) at Sable Island, Canada, between 1995 and 2018, we tested the hypothesis that differences in foraging behavior and diet prior to the breeding season, estimated through satellite telemetry, stable isotope (SI) signatures and quantitative fatty acid signature analysis (QFASA), respectively, affect reproductive success. Females consuming a diet of lower diversity and with more positive δ13C estimates, possibly indicating more benthic foraging, had higher maternal postpartum mass; however, metrics of maternal foraging behavior and diet alone did not directly influence pup weaning mass.

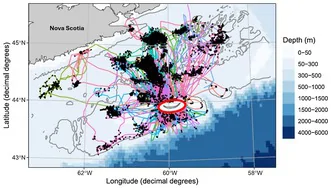

## 1 Introduction

Understanding how animals interact with their environment and how spatial and temporal variation in foraging behavior influences reproductive success and population dynamics is an important area in ecology (Nathan et al. 2008; Hays et al. 2016). Nevertheless, our knowledge of how foraging behavior influences individual fitness in free‐ranging animals remains limited (Gaillard et al. 2010; Whitlock et al. 2015; Bowen and Jonsen 2022). The relationships among foraging effort, diet, and fitness are often studied within the framework of optimal foraging theory, which predicts that animals make movement decisions and select prey that optimize their net energy intake (MacArthur and Pianka 1966; Schoener 1971; Pyke 1984). Thus, exploring the consequences of individual variation in foraging effort and diet composition should improve our understanding of how foraging decisions influence individual survival and fitness (Johnson 1980; Manly et al. 2002). However, such studies are uncommon since obtaining simultaneous data on foraging effort, diet, and reproductive success of individual free‐ranging animals is difficult, particularly for marine predators (Whitlock et al. 2015).

Vertebrates fuel the costs of reproduction along a continuum between income and capital breeding strategies, whereby individuals that use an income breeding strategy rely on concurrent energy accumulation during the breeding period to support the costs of reproduction while capital breeding individuals expend energy acquired and stored prior to the breeding period to support reproductive costs (Stephens et al. 2009, 2014). A capital breeding strategy is thought to be advantageous for large animals capable of carrying large energy stores since it offers energetic benefits in areas of patchy or unreliable food availability and allows parents to direct more resources (e.g., time and energy) to offspring, as opposed to foraging during the offspring rearing period (Stephens et al. 2009, 2014). A capital breeding strategy is particularly advantageous for many phocid species, allowing them to utilize extended foraging periods over large spatial and temporal scales to build up energy reserves for relatively short, but intense, breeding and lactation periods (Costa and Maresh 2022; Avery and Zinn 2023).

Links between foraging behavior and reproductive success have been studied in several marine predators. Among income‐breeding marine seabirds, foraging site fidelity in females is positively correlated with reproductive success in the Black‐browed Albatross ( *Thalassarche melanophris* ; Patrick and Weimerskirch 2017). In the European Shag ( *Phalacrocorax aristotelis* ), reproductive success is closely tied with the availability of their main prey source, saithe ( *Pollachius virens* ), such that annual foraging trip distance decreases and reproductive success increases as the proportion of saithe in the diet increases (Lorentsen et al. 2019). Similarly, increased reproductive success in lactating Antarctic fur seals ( *Arctocephalus gazella* ), an income breeder, is associated with greater foraging efficiency (Jeanniard‐du‐Dot et al. 2017). Among capital breeding marine mammals, a period of reduced reproductive output in the South African population of the southern right whale ( *Eubalaena australis* ; van den Berg et al. 2020) was correlated with a long‐term shift in stable isotopes of carbon and nitrogen, increased variability in foraging behavior, and declining prey abundance. Foraging success is also an important predictor of reproductive success in Pacific gray whales ( *Eschrichtius robustus* ; Perryman et al. 2021). Within capital breeding phocids, studies on the southern (Vergani et al. 2008; McMahon et al. 2017) and northern (Crocker et al. 2006; Robinson et al. 2012) elephant seals ( *Mirounga leonina* and *M. angustirostris* , respectively) have demonstrated that favorable environmental conditions and increased foraging success are linked to increased natality rates, maternal mass on return to the breeding site, and pup weaning mass (PWM) and survival. Taken together, data from diverse marine predators demonstrate that the foraging behavior of individuals has both short‐ and longer‐term consequences for reproductive success.

In the context of the bigger‐is‐better hypothesis (Ralls 1976), a broad relationship between maternal body mass at parturition, a proxy for energy stores, and reproductive success has been established in both capital (gray seals *Halichoerus grypus*, Iverson et al. 1993; Mellish et al. 1999; Bowen et al. 2015; northern elephant seals, Robinson et al. 2012; Le Boeuf et al. 2019; Beltran et al. 2023; southern elephant seals, McMahon et al. 2017; polar bears *Ursus maritimus* , Atwood et al. 2021; bighorn sheep *Ovis canadensis*, Stephenson et al. 2020 and white‐tailed deer *Odocoileus virginianus* , Michel et al. 2019) and income breeding species (Wandering Albatross *Diomedea exulans* , Weimerskirch 2018; Rockhopper Penguins *Eudyptes chrysocome* , Dehnhard et al. 2015; and European Shag, Lorentsen et al. 2019). Given the importance of pre‐breeding foraging success and energy acquisition in capital breeding marine mammals, these species offer interesting model systems to study the relationships between foraging effort, diet, and reproductive success.

Phocid seals are generally wide‐ranging, large‐bodied, and long‐lived marine predators foraging across a range of habitats and temporal scales (Bowen and Jonsen 2022). Gray seals are a capital breeding, sexually dimorphic, and iteroparous phocid species. In the northwest Atlantic, they inhabit the continental shelf from the Gulf of Maine north to southern Labrador (Breed et al. 2006, 2009). Females reach sexual maturity at age 4–5 years and can continue to reproduce into their early 40s (Bowen et al. 2006). Pregnant females haul out on Sable Island in December through early February to give birth to a single pup that they nurse for 16–18 days (Bowen et al. 2006). Females mate during late lactation and return to sea to replenish body reserves after abruptly weaning their pup. Weaned pups fast for several weeks relying on the energy acquired during lactation before undertaking their first foraging trip (Noren et al. 2008).

While pinniped pup fitness can be influenced by multiple factors, including maternal contaminant transfer (e.g., Debier et al. 2003; Vanden Berghe et al. 2012; Habran et al. 2013; Peterson et al. 2024) and immune status and susceptibility to disease (e.g., Hall et al. 2002; Baily et al. 2024), here we focus on the relationships between a female's foraging effort and diet, her body mass at parturition (maternal postpartum mass, MPPM), and the body mass of her pup at weaning (PWM). Lactating females rely solely on the energy stores accumulated during the foraging period prior to parturition to support the energetic costs of their own metabolism and milk production, resulting in an average loss of 37% of their postpartum body mass (Iverson et al. 1993; Sanchez et al. 2026). Maternal mass loss is a good predictor of pup growth rates during lactation, with heavier mothers at parturition weaning heavier pups (Iverson et al. 1993; Mellish et al. 1999). At the Sable Island colony, pup survival is positively related to pup weaning mass up to the mean weaning mass of 51.5 kg (Bowen et al. 2015). A similar relationship between pup weaning mass and survival has also been documented in UK gray seals (Hall et al. 2001). Given the importance of maternal energy acquisition in supporting lactation, understanding the consequences of variation in foraging effort and diet should improve our understanding of the factors influencing reproductive success. Further, there is evidence from Baltic gray seals that the blubber thickness of adult females (Kauhala et al. 2017, 2019), as well as birth rates (Kauhala et al. 2016), are related to the quality (i.e., weight) of preferred prey species available, suggesting that the availability and acquisition of high quality prey could be an important driver of reproductive success in this species.

In this paper, we tested the hypothesis that differences in foraging effort and diet influence the ability of individual females to store energy prior to parturition which, in turn, influences their reproductive success. We first used data on the movements of individual females estimated from satellite telemetry collected prior to the breeding season, and their diets during the same period estimated using stable isotope (SI) signatures and quantitative fatty acid (FA) signature analysis (QFASA) to predict her MPPM. We then tested for relationships between estimated foraging effort or diet characteristics and her PWM to explore maternal foraging and diet effects on offspring after accounting for MPPM.

## 2 Materials & Methods

### 2.1 Satellite Tag Deployments and Data Collection

Our study was conducted on Sable Island, a crescent shaped sandbar, located ~300 km off Halifax, Nova Scotia, Canada, which is home to the largest breeding colony of gray seals in the world (Bowen et al. 2007; den Heyer et al. 2021; Figure 1). Between 1995 and 2018, 60 adult female gray seals were captured on land in June, September and October on Sable Island using hand‐held nets and weighed to the nearest 0.5 kg. Females were then anesthetized using Telazol (0.90 mg kg^−1^; Zoetis Canada Inc.) to allow accurate measurement of total body length (to the nearest cm) and attachment of the instruments to the animal's neck pelage using 5‐min epoxy (Austin et al. 2003). Transmitters weighed an average of ~0.3% of female body mass. Justrabo et al. (2024) found no evidence that these externally mounted instruments negatively influence female mass gain prior to parturition or subsequent female reproductive performance. All study females were of known‐age as they had been marked with a unique, permanent hot‐iron brand shortly after they were weaned as pups (Bowen et al. 2006).

**FIGURE 1:**
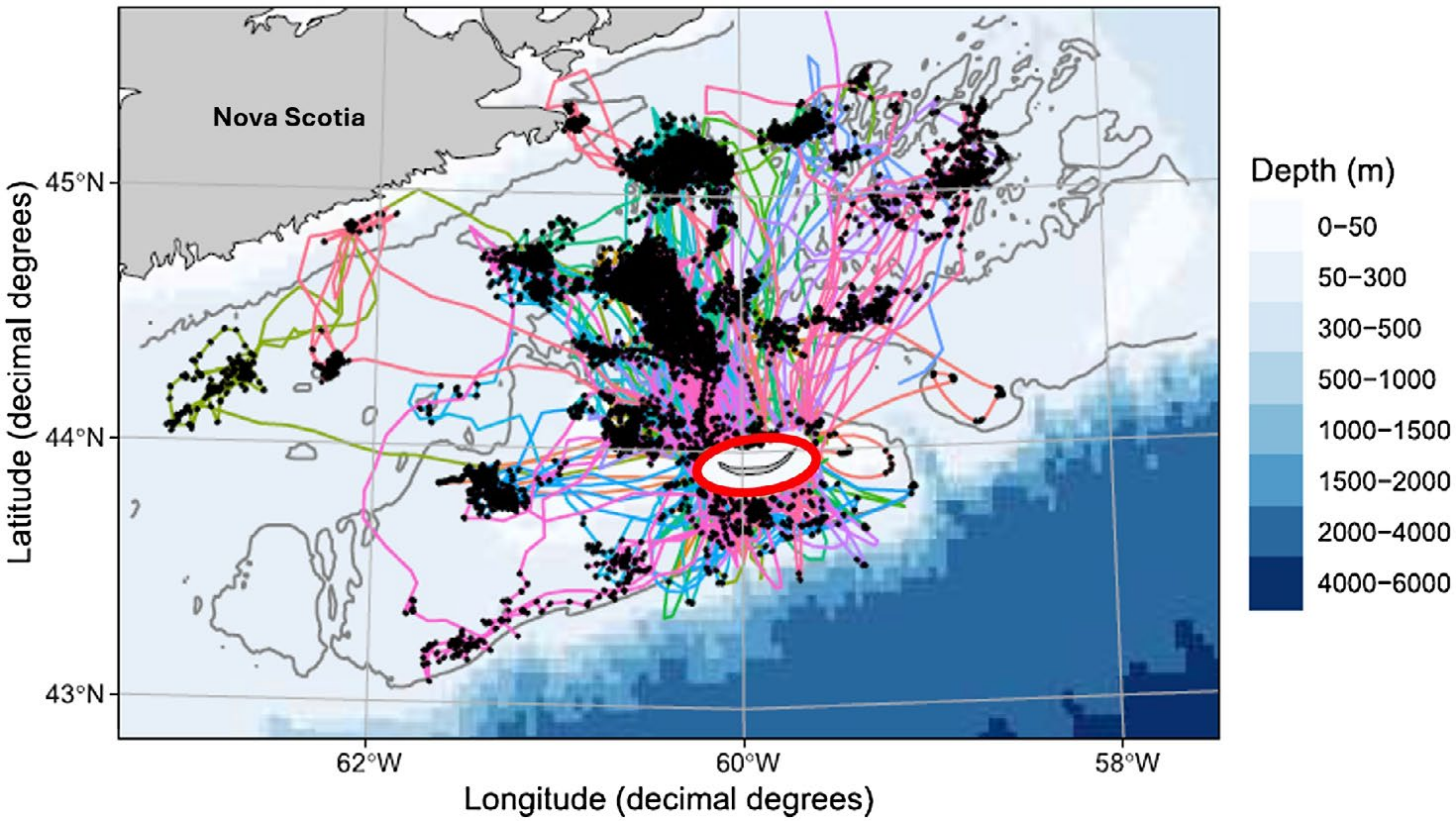
Hidden Markov model (HMM) inferred foraging locations (black points) and movement tracks over the Scotian Shelf from 42 female gray seal ( *Halichoerus grypus* ) deployments during the fall foraging period between 1995 and 2018. Each deployment is represented by a uniquely colored movement track. Sable Island is circled in red. Bathymetric data were acquired from the NOAA ETOPO1 database (Amante and Eakins 2009).

All 60 study females returned to Sable Island during the subsequent December–February breeding season and were located using their VHF transmitter signal and/or visually during an annual winter mark‐resighting and reproductive ecology program (Badger et al. 2023). Given that all females in this study successfully birthed a pup, our sample consists of adult females in sufficient condition to carry pregnancy to term. When a female was first located with her newborn pup, she was left undisturbed for 3 days to allow for the mother‐pup bond to form. At three or more days postpartum, the female and pup were captured using hand‐held nets and weighed (pups to the nearest 0.5 kg) and pup sex was recorded. These weights were used as proxies for MPPM and pup birth mass. Full‐depth blubber biopsies (*n* = 42) taken from the posterior flank of adult females and skin samples from their rear flipper (*n* = 40) were collected for FA and SI diet analysis, respectively, following the methods described in Beck et al. (2007) and Tucker et al. (2008). Prior to release, pups were tagged with uniquely numbered tags in the hind flipper to ensure accurate identification after the female weaned her pup and returned to sea, leaving her pup in the colony.

### 2.2 Location Data Processing and Hidden Markov Model (HMM) Estimation of Behavioral States

Data processing and analyses were performed using R 4.3.1 (R Core Team 2023). Locations were estimated from satellite‐linked data transmitted to the Argos satellite system or from high‐resolution GPS locations stored by the MK10‐AF Fastloc transmitters. MK10‐AF Fastloc instruments were used in deployments from 2009 onward and these higher‐resolution GPS locations were used in analyses when available (see Henry‐Adams et al., submitted).

GPS locations were initially filtered to remove inaccurate locations following (Dujon et al. 2014; Lidgard et al. 2014). The remaining locations were then filtered using the *trackfiltering* R package (Lawler and Mills Flemming, in peer review) to remove additional inaccurate locations and to temporally regularize observations. The true locations were initially modeled as a Gaussian process with each location having an observation error, with the longitude and latitude components of the location coordinates modeled separately. While the exact location error is not provided on Argos locations, each observation is assigned an Argos quality error class based on the number of messages received on the satellite pass and the estimated error. GPS locations are assumed to have minimal error and to be more accurate than Argos Class 3 locations (Dujon et al. 2014; Lawler and Mills Flemming, in peer review).

Of the 60 deployments, 42 had high quality movement tracks suitable for further analyses. Of the 42 with high quality tracks, five collected Argos locations between 1995 and 2004, and 37 collected GPS locations between 2009 and 2018. Tracks were regularized to a six‐hour timestep and any tracks that crossed land were rerouted to the closest seawater using the R package *pathroutr* (London 2020; Henry‐Adams et al., submitted). While we acknowledge the difference in spatial accuracy and temporal resolution between Argos only and Fastloc‐GPS derived location data, careful consideration was given to the time step chosen and movement tracks selected for analysis such that the estimated behavioral states from these data types were comparable. The six‐hour timestep was selected to ensure that the average location transmission frequency was equal to or greater than the chosen time step.

We used the R package *MomentuHMM* (McClintock and Michelot 2018) to fit a two‐state Hidden Markov model (HMM) to the processed location data for the 42 high‐quality movement tracks. Separate HMMs were fit to the GPS and Argos‐based tracks (see Henry‐Adams et al., submitted, for details). Two latent states, proxies for behavioral states, used in this model were “directed traveling”, which is characterized by large step lengths and high directional persistence, and “putative foraging”, which is characterized by area‐restricted search behavior with shorter step lengths and low directional persistence.

The tracks of individual females were truncated such that we only used locations received after September 1st. We did this so that estimates of foraging effort were temporally relevant with their associated diet estimates and consistent across all individuals. Following Breed et al. (2009), individual tracks were separated into foraging trips and trips that were less than 24 h in length were removed, resulting in 94 locations (or 0.8%) of all locations being removed. Bathymetric data was acquired from the NOAA ETOPO1 database (Amante and Eakins 2009) via the R package *marmap* (Pante and Simon‐Bouhet 2013). We then calculated the total number of foraging patches used across all foraging trips of an individual, where a foraging patch was defined as having at least three consecutive putative foraging locations, again per Breed et al. (2009). The total distance traveled by an individual across all foraging trips and the proportion of deployment time spent in a putative foraging state were also calculated. Foraging areas were identified and named according to the topographical feature over which foraging took place (Figure 1) given that one or more area‐restricted searches (ARSs) were identified at that location. Initially, we intended to test whether foraging areas were related to MPPM and PWM. However, the combination of a limited sample size and a large imbalance in the number of observations across areas used prevented us from making meaningful conclusions from these analyses.

### 2.3 Diet Estimation

For 42 of the 60 deployments, diets were estimated using QFASA following the methods described in Stewart et al. (2022) for FA samples collected during the breeding season (December–February, winter). These samples reflected diet in the several months leading up to the sampling period (Iverson et al. 2004). Prey species included in the QFASA estimation are given in Stewart et al. (2022). Details of the chemical processing of FA and SI samples are given in Budge et al. (2006) and Tucker et al. (2008), respectively. Energy density of the estimated diet of each individual was calculated following Beck et al. (2007). Diet diversity was calculated using a standardized Shannon–Weiner Index (Krebs 1989; Newton‐Fisher 1999). We also collected skin samples from 40 of the deployments for SI analysis at instrument recovery. SI analysis was performed as described in Tucker et al. (2007). Stable carbon and nitrogen ratios are expressed in delta (δ) notation, expressed as the parts per thousand (‰) deviation from a standard material. δ^13^C values were corrected for known fractionation rates for seal skin tissue.

We used a k‐means cluster analysis without a pre‐determined number of clusters to group individual diets based on compositional similarities. Since QFASA diet estimates are compositional in nature, a centered log‐ratio transformation was applied to the data before cluster analysis. Two diet clusters were identified based on an elbow plot method and silhouette analysis (Figure A2). A cluster analysis was then performed to sort individual diets into one of the two identified groups. All species identified as composing ≥ 5% of the diet, on average, of either diet group were then used to describe each group (Figure A3). Further information on the calculation of diet characteristics is provided in Appendix A.

### 2.4 Measuring Reproductive Success

During the breeding season, 60 females and their pups were first sampled “at birth” (i.e., 3–5 days postpartum used as proxy for birth mass), as described previously (see *Satellite Tag Deployments and Data Collection*). Since females attend their pups continuously throughout lactation on Sable Island, once a pup was sighted alone, it was considered weaned and was captured and weighed to the nearest 0.5 kg. Because the initial deployment dates in summer and fall differed greatly among females and rates of mass gain vary seasonally (Beck et al. 2003, 2003a, 2003b), we used MPPM as the measure of female energy acquisition rather than female mass gain over the foraging period leading up to parturition. Differences in MPPM have previously been shown to directly reflect differences in energy stores at parturition and reproductive performance in gray seals (Iverson et al. 1993; Mellish et al. 1999; Pomeroy et al. 1999).

Over the course of lactation, females lose an average of 4.1 kg/day of body mass (Mellish et al. 1999; Sanchez et al. 2026). Given that not all returning females were located on day three postpartum (due to inclement weather) and that females lose significant mass daily, we included pup developmental stage (ranging from one—newborn to three—fat white pup) at the time of measuring MPPM in all models. The sex of the pup was included in all models to account for the fact that male pups are on average larger than female pups (Bowen et al. 2006, 2020) and females have been shown to invest more in male pups compared to female pups (Anderson and Fedak 1987). In total, MPPM was collected from 60 females where pup sex and birthdate or a proxy for birthdate (stage at first sighting) were known and associated estimates of foraging effort and/or diet were available. PWM was known for 36 of these 60 females.

### 2.5 Statistical Analysis

The influence of diet characteristics and estimated foraging effort on MPPM and PWM was tested using generalized linear models (GLMs) fit using the R package *stats* (R Core Team 2023). To normalize covariates and make coefficients and effect sizes comparable, diet diversity, energy density, δ^13^C, δ^15^N, deployment duration, and all mass and length values were standardized to a mean = 0 and standard deviation = 1.

Estimates of foraging effort, diet, and maternal and pup masses were not available for all females. Therefore, we examined three separate suites of models using subsets of the 60 females to relate estimated foraging effort, QFASA estimated diet, or SI estimated diet to MPPM and PWM, respectively. We did this to maximize the sample size for each suite of models. To relate covariates to MPPM, we fit the following GLMs using a Gaussian error distribution and log link function:

(1) $$\begin{array}{c} {\text{MPPM}}_{i,t}=\beta_{0}+\beta_{1}\cdot {\text{MomAge}}_{i,t}+\beta_{2}\cdot {{\text{MomAge}}^{2}}_{i,t}+\beta_{3}\cdot {\text{Length}}_{i,t}\\ \;+\beta_{4}\cdot {\text{PupStage}}_{i,t}+\beta_{5}\cdot {\text{PupSex}}_{i,t}+\beta_{6}\cdot {\text{Duration}}_{i,t}+\beta_{7}\cdot \%{\text{For}}_{i,t}\\ \;+\beta_{8}\cdot {\text{Dist}}_{i,t}+\beta_{9}\cdot {\text{ForPatch}}_{i,t} \end{array}$$

(2) $$\begin{array}{c} {\text{MPPM}}_{i,t}=\beta_{0}+\beta_{1}\cdot {\text{MomAge}}_{i,t}+\beta_{2}\cdot {{\text{MomAge}}^{2}}_{i,t}+\beta_{3}\cdot {\text{Length}}_{i,t}\\ \;+\beta_{4}\cdot {\text{PupStage}}_{i,t}+\beta_{5}\cdot {\text{PupSex}}_{i,t}\;+\beta_{6}\cdot {\mathrm{Eng}}_{i,t}+\beta_{7}\cdot {\mathrm{Div}}_{i,t}\\ \;+\beta_{8}\cdot {\text{Group}}_{i,t} \end{array}$$

(3) $$\begin{array}{c} {\text{MPPM}}_{i,t}=\beta_{0}+\beta_{1}\cdot {\text{MomAge}}_{i,t}+\beta_{2}\cdot {{\text{MomAge}}^{2}}_{i,t}+\beta_{3}\cdot {\text{Length}}_{i,t}\\ \;+\beta_{4}\cdot {\text{PupStage}}_{i,t}+\beta_{5}\cdot {\text{PupSex}}_{i,t}+\beta_{6}\cdot \delta^{13}C_{i,t}+\beta_{7}\cdot \delta^{15}N_{i,t,} \end{array}$$

To examine variation in PWM, we fit the following GLMs using a gamma error distribution and log link function:

(4) $$\begin{array}{c} {\mathrm{PWM}}_{i,t}=\beta_{0}+\beta_{1}\cdot {\text{MomAge}}_{i,t}+\beta_{2}\cdot {{\text{MomAge}}^{2}}_{i,t}+\beta_{3}\cdot {\text{MPPM}}_{i,t}\\ \;+\beta_{4}\cdot {\text{PupStage}}_{i,t}+\beta_{5}\cdot {\text{PupSex}}_{i,t}\;+\beta_{6}\cdot {\text{Duration}}_{i,t}\\ \;+\beta_{7}\cdot \%{\text{For}}_{i,t}+\beta_{8}\cdot {\text{Dist}}_{i,t}+\beta_{9}\cdot {\text{ForPatch}}_{i,t} \end{array}$$

(5) $$\begin{array}{c} {\mathrm{PWM}}_{i,t}=\beta_{0}+\beta_{1}\cdot {\text{MomAge}}_{i,t}+\beta_{2}\cdot {{\text{MomAge}}^{2}}_{i,t}+\beta_{3}\cdot {\text{MPPM}}_{i,t}\\ \;+\beta_{4}\cdot {\text{PupStage}}_{i,t}+\beta_{5}\cdot {\text{PupSex}}_{i,t}+\beta_{6}\cdot {\mathrm{Eng}}_{i,t}+\beta_{7}\cdot {\mathrm{Div}}_{i,t}\\ \;+\beta_{8}\cdot {\text{Group}}_{i,t} \end{array}$$

(6) $$\begin{array}{c} {\mathrm{PWM}}_{i,t}=\beta_{0}+\beta_{1}\cdot {\text{MomAge}}_{i,t}+\beta_{2}\cdot {{\text{MomAge}}^{2}}_{i,t}+\beta_{3}\cdot {\text{MPPM}}_{i,t}\\ \;+\beta_{4}\cdot {\text{PupStage}}_{i,t}+\beta_{5}\cdot {\text{PupSex}}_{i,t}\;+\beta_{6}\cdot \delta^{13}C_{i,t}+\beta_{7}\cdot \delta^{15}N_{i,t} \end{array}$$

where *β*_0_ is the intercept, parameters *β*_1_–_9_ are the regression coefficients for covariates, %For is the scaled estimated proportion of deployment time spent in a putative foraging state, Dist is the scaled total distance traveled, and ForPatch is the number of foraging patches (ARS patterns) for individual *i* in year *t*, Eng and Div are the scaled estimates of energy density and diversity of the diet of individual *i* in year *t*, Group is the assigned diet group (redfish dominant or sand lance dominant diet; Appendix A) of individual *i* in year *t* based on cluster analysis (see Henry‐Adams et al., submitted), δ^13^C and δ^15^N are the standardized carbon and nitrogen ratio estimates of individual *i* in year *t*, and Duration is the scaled value for the number of days between the first location obtained after September 1st in year *t* and the last location before the female hauled out for the December–February breeding season. Although total foraging trip duration was calculated as a metric of foraging effort, it was excluded from models due to high correlation (*r*_pearson_ = 0.81) with total distance traveled. Although both metrics relate to foraging effort, we elected to retain total distance traveled rather than trip duration due to its more direct relationship with foraging range and energetic output, which are commonly rooted in animal foraging theory (e.g., Charnov 1976; Ford 1983; Birt et al. 1987). Deployment duration was included as a covariate to account for differing durations between the first location obtained after September 1st and the last location before the female hauled out for the breeding season. Linear and quadratic terms for maternal age (MomAge) at each observation were included in all models to account for previously observed differences in reproductive performance with age (Bowen et al. 2006). Female body length was included as a covariate to account for female body size when examining the effects of estimated foraging effort or diet on MPPM. Since heavier females produce heavier pups at weaning (Iverson et al. 1993; Mellish et al. 1999), MPPM was included in models relating covariates to PWM. For the model structures outlined above, 42 observations were available for model structures one and two, 40 for model structure three, and 36 for model structures four‐six.

Global models were fit before stepwise AIC variable selection, as well as log likelihood ratio comparisons, were used to identify preferred models explaining each response variable (Tables 2 and 3). Akaike weights (*w*_i_) were also compared between candidate model structures to evaluate AIC evidence ratios and AIC model comparisons. Although two females were deployed in two largely separated study years (5 and 9 years apart, respectively), independence in observations is assumed as the number of repeated measurements was not large enough to estimate a random effect of individual ID. Due to a limited number of observations in some study years, our models were unable to estimate a random effect of year, meaning the current dataset is unable to test for and assumes no effect of study year on the response variables.

## 3 Results

The age of the 60 females with MPPM and associated pup data ranged from 11 to 31 years with an average age of 24.1 years (Table 1). The total length of these females ranged from 167 to 201 cm with an average MPPM of 193.9 ± 29.0 kg. These females produced 35 male pups with an average PWM of 51.7 ± 6.6 kg and 25 female pups with an average PWM of 50.4 ± 7.35 kg.

**TABLE 1:** Summary statistics of foraging effort (*n* = 42), diet (QFASA estimated; *n* = 42, and SI estimated δ^13^C and δ^15^N; *n* = 40) and maternal and pup traits from 60 mother‐pup pairs.

Measure	Mean	Median	Standard deviation	Range
**Foraging effort**				
Total distance traveled (km)	1719.9	1757.7	569.5	(462.4, 2759.2)
% of deployment in foraging state	56.9	57.6	11.2	(28.7, 75.2)
Number of foraging patches	7.9	7.5	3.3	(2, 16)
Deployment duration	100.8	109.0	20.5	(54, 128)
Total trip duration (days)	75.6	78.8	18.2	(38.5, 104.8)
**Diets**				
Diet diversity	0.34	0.35	0.1	(0.1, 0.5)
Energy density (kJ/g of prey tissue)	5.8	5.8	0.2	(5.2, 6.5)
δ^13^C (‰)	−17.0	−17.0	0.4	(−17.7, −16.2)
δ^15^N (‰)	17.3	17.3	0.5	(16.0, 18.2)
Diet group (number of individuals)	Redfish dominant		Sand lance dominant	
	18		24	
**Reproductive performance**				
Maternal Postpartum Mass (MPPM, kg)	193.9	191.2	29.0	(130.0, 252.0)
Maternal length (cm)	185.8	186.5	8.0	(167.0, 201.0)
Maternal age (years)	24.1	25.5	4.9	(11.0, 31.0)
Pup weaning mass (PWM, kg)	51.2	51.5	6.8	(34.5, 68.0)
Pup stage at MPPM measurement	1.7	1.5	0.8	(1, 4)
Pup sex (number of individuals)	Male		Female	
	35		25	

Data from satellite‐linked transmitters yielded foraging estimates for 42 females covering an average of 101 days leading up to the December–February breeding season. During this period, females traveled an estimated average of 1720 km and spent 56.9% of deployment time in a putative foraging state (Table 1). Most foraging was focused over the central and eastern Scotian Shelf (Figure 1), however, one individual spent large portions of the fall foraging period in the Gulf of St. Lawrence.

During the fall foraging period leading up to parturition, females had an estimated diet diversity of 0.34 ± 0.1 and a diet energy density of 5.8 ± 0.2 kJ/g (Table 1). Based on this analysis, all individuals were grouped into one of two diet groups, classified as having either a redfish‐dominant diet or a diet primarily composed of northern sand lance (Appendix A). Within the redfish diet group, individuals consumed an average of 61% redfish and about 8% sand lance, whereas in the sand lance dominant diet group, sand lance and redfish made up about 43% and 27% of the diet, respectively. SI analysis revealed that the overall δ^13^C and δ^15^N ratios across all individuals where skin samples were acquired averaged −17.0 ± 0.4 (‰) and 17.3 ± 0.5 (‰), respectively.

The models we examined to account for variation in MPPM and PWM are given in Tables 2 and 3, respectively. The best supported foraging model explaining MPPM included total distance traveled by females and the duration of the movement track, along with pup stage and sex and maternal age and body length. Although distance was retained, it was not a significant predictor of MPPM. However, there was a significant positive relationship between duration of deployment and MPPM (Table 4). The best supported QFASA diet model retained diet diversity along with maternal age and body length to explain variation in MPPM (Table 5). Our data indicated that diet diversity was inversely related to MPPM (*p* < 0.001; R^2^ = 0.46, Figure 2). There was weak support for diet diversity along with a quadratic form of maternal age, but the first model was twice as likely according to AIC weights (Table 2). Neither diet cluster nor energy density of the diet were useful predictors of variation in MPPM. Models using only SI data indicated that variation in MPPM was best explained by levels of both carbon and nitrogen isotopes along with female body length (Table 6). Although retained in the model, δ^15^N level was not a useful predictor. There was a positive relationship between δ^13^C and MPPM suggesting that the habitat used by females influenced foraging success (*p* < 0.001; R^2^ = 0.42, Figure 3).

**TABLE 2:** Summary of GLMs relating estimates of foraging effort (*n* = 42), QFASA estimated diet characteristics (*n* = 42), and SI estimates (*n* = 40) to maternal postpartum mass (MPPM) from female gray seal ( *Halichoerus grypus* ) deployments between 1995 and 2018.

Foraging model	df	LL	AIC_c_	ΔAIC_c_	*w*_i_
**MPPM ~ Dist + Duration + PupStage + PupSex + MomAge + Length**	8	−178.06	376.49	0	0.80
MPPM ~ %For + Dist + Duration + PupStage + PupSex + MomAge + Length	9	−178.03	379.69	3.20	0.16
MPPM ~ %For + Dist +Duration + PupStage + PupSex + MomAge + MomAge^2^ + Length	10	−178.00	383.10	6.61	0.04
MPPM ~ %For + Dist + ForPatch + Duration + PupStage + PupSex + MomAge + MomAge^2^ + Length	11	−177.99	386.78	10.29	0
MPPM~1	2	−202.36	409.02	32.53	0
**QFASA Diet Model**					
**MPPM ~ Div + MomAge + Length**	5	−177.95	367.56	0	0.55
MPPM ~ Div + MomAge + MomAge^2^ + Length	6	−177.24	368.88	1.32	0.28
MPPM ~ Div + Group + MomAge + MomAge^2^ + Length	7	−176.61	370.52	2.96	0.13
MPPM ~ Div + Group + PupSex + MomAge + MomAge^2^ + Length	8	−176.44	373.25	5.69	0.03
MPPM ~ Div + Group + PupStage + PupSex + MomAge + MomAge^2^ + Length	9	−176.44	376.51	8.95	0.01
MPPM ~ Div + Eng + Group + PupStage + PupSex + MomAge + MomAge^2^ + Length	10	−176.44	379.98	12.42	0
MPPM ~1	2	−190.4	385.12	17.56	0
**Sable Isotope Model**					
**MPPM ~ δ** ^**13**^ **C + δ** ^**15**^ **N + Length**	5	−173.15	358.07	0	0.67
MPPM ~ δ^13^C + δ^15^N + MomAge + Length	6	−172.99	360.15	2.08	0.24
MPPM ~ δ^13^C + δ^15^N + PupStage + MomAge + Length	7	−172.55	362.61	4.54	0.07
MPPM ~ δ^13^C + δ^15^N + PupStage + MomAge + MomAge^2^ + Length	8	−172.45	365.55	7.48	0.02
MPPM ~ δ^13^C + δ^15^N + PupStage + PupSex + MomAge + MomAge^2^ + Length	9	−172.40	368.81	10.74	0
MPPM~1	2	−184.02	372.36	14.29	0

*Note:* The preferred model for each foraging characteristic is in bold font. The number of degrees of freedom (df), log‐likelihood (LL), second‐order Akaike information criterion (AIC_c_), ΔAIC_c_, and Akaike weight (*w*_i_) are listed.

**TABLE 3:** Summary of all GLMs relating estimates of foraging effort (*n* = 36), QFASA estimated diet characteristics (*n* = 36), and SI estimates (*n* = 36) to pup weaning mass (PWM) from female gray seal ( *Halichoerus grypus* ) deployments occurring between 1995 and 2018.

Foraging model	df	LL	AIC_c_	ΔAIC_c_	*w*_i_
**PWM ~ Dist + MPPM + PupStage + MomAge**	6	−106.85	228.60	0	0.61
PWM ~ Dist + ForPatch + MPPM + PupStage + MomAge	7	−106.24	230.47	1.87	0.24
PWM ~ Dist + ForPatch + MPPM + PupStage + MomAge + MomAge^2^	8	−105.48	232.39	3.79	0.10
PWM ~ Dist + ForPatch + MPPM + PupStage + PupSex + MomAge + MomAge^2^	9	−104.49	233.89	5.29	0.04
PWM ~ %For + Dist + ForPatch + MPPM + PupStage + PupSex + MomAge + MomAge^2^	10	−104.35	237.49	8.89	0.01
PWM ~ %For + Dist + ForPatch + Duration + MPPM + PupStage + PupSex + MomAge + MomAge^2^	11	−104.32	241.63	13.03	0
PWM~1	2	−118.95	242.26	13.66	0
**QFASA Diet Model**					
**PWM ~ MPPM + MomAge**	4	−106.86	223.01	0	0.53
PWM ~ Div + MPPM + MomAge	5	−106.05	224.11	1.10	0.31
PWM ~ Div + Eng + MPPM + MomAge	6	−105.59	226.08	3.07	0.12
PWM ~ Div + Eng + MPPM + PupStage + MomAge	7	−105.29	228.59	5.58	0.03
PWM ~ Div + Eng + MPPM + PupStage + PupSex + MomAge	8	−105.09	231.51	8.50	0.01
PWM ~ Div + Eng + Group + MPPM + PupStage + PupSex + MomAge	9	−105.00	234.92	11.91	0
PWM ~ Div + Eng + Group + MPPM + PupStage + PupSex + MomAge + MomAge^2^	10	−104.96	238.72	15.71	0
PWM~1	2	−119.38	243.13	20.12	0
**Sable Isotope Model**					
**PWM ~ MPPM + MomAge**	4	−101.92	213.17	0	0.54
PWM ~ MPPM + PupStage + MomAge	5	−101.20	214.46	1.29	0.29
PWM ~ MPPM + PupStage + PupSex + MomAge	6	−100.55	216.10	2.93	0.12
PWM ~ MPPM + PupStage + PupSex + MomAge + MomAge^2^	7	−100.25	218.65	5.48	0.04
PWM ~ δ^13^C + MPPM + PupStage + PupSex + MomAge + MomAge^2^	8	−100.20	221.93	8.76	0.01
PWM ~ δ^13^C + δ^15^N + MPPM+ PupStage + PupSex + MomAge + MomAge^2^	9	−100.15	225.50	12.33	0
PWM~1	2	−119.28	242.92	29.75	0

*Note:* The preferred model for each foraging characteristic is in bold font. The number of degrees of freedom (df), log‐likelihood (LL), second‐order Akaike information criterion (AIC_c_), ΔAIC_c_, and Akaike weight (*w*_i_) are listed.

**TABLE 4:** Summary of the coefficient estimates (Est) and standard error (Std. Error) for the scaled covariates of models with > 0.20 Akaike weight relating estimated foraging characteristics (dist = total distance traveled; ForPatch = the number of foraging patches used) to maternal postpartum mass (MPPM; *n* = 42) and pup weaning mass (PWM; *n* = 36) from female gray seal ( *Halichoerus grypus* ) deployments taking place between 1995 and 2018.

Model	Dist		ForPatch		Duration		PupSex		PupStage		MomAge		Length		MPPM	
	Est.	Std. Error	Est.	Std. Error	Est.	Std. Error	Est.	Std. Error	Est.	Std. Error	Est.	Std. Error	Est.	Std. Error	Est.	Std. Error
**MPPM ~ Dist + Duration + PupSex + PupStage + MomAge + Length**	−0.05	0.03	—	—	0.06*	0.03	0.06	0.03	−0.09*	0.02	0.07*	0.02	0.06*	0.02	X	X
**PWM ~ Dist + MPPM + PupStage + MomAge**	−0.04	0.03	—	—	—	—	—	—	0.07*	0.02	−0.04	0.03	X	X	0.11*	0.02
PWM ~ Dist + ForPatch + MPPM + PupStage + MomAge	−0.03	0.03	−0.01	0.01	—	—	—	—	0.06*	0.03	−0.03	0.03	X	X	0.11*	0.02

*Note:* Asterisks indicate significance at *p* < 0.05. An “X” indicates that the covariate was not included in the particular model. Models with the highest evidence ratio for each response variable is in bolded font. The model selection tables are presented in Tables 2 and 3.

**TABLE 5:** Summary of the preferred models with > 0.20 Akaike weight relating QFASA estimated diet characteristics to maternal postpartum mass (MPPM; *n* = 42) and pup weaning mass (PWM; *n* = 36) from female gray seal ( *Halichoerus grypus* ) deployments taking place between 1995 and 2018.

Model	Diet diversity		MomAge		MomAge^2^		Length		MPPM	
	Est.	Std. Error	Est.	Std. Error	Est.	Std. Error	Est.	Std. Error	Est.	Std. Error
**MPPM ~ DietDiversity + MomAge + Length**	−0.06*	0.02	0.04*	0.02	—	—	0.03*	0.01	X	X
MPPM ~ DietDiversity + MomAge + MomAge^2^ + Length	−0.05*	0.02	0.06*	0.02	0.02	0.02	0.04*	0.02	X	X
**PWM ~ MomAge + MPPM**	—	—	−0.07*	0.02	—	—	X	X	0.12*	0.02
PWM ~ DietDiversity + MomAge + MPPM	−0.03	0.02	−0.06*	0.02	—	—	X	X	0.11*	0.02

*Note:* Coefficient estimates and standard errors for the scaled covariates are provided. Asterisks indicate significance at *p* < 0.05. An “X” indicates that the covariate was not included in the particular model. Models with the highest evidence ratio for each response variable are in bolded font. The model selection tables are presented in Tables 2 and 3.

**FIGURE 2:**
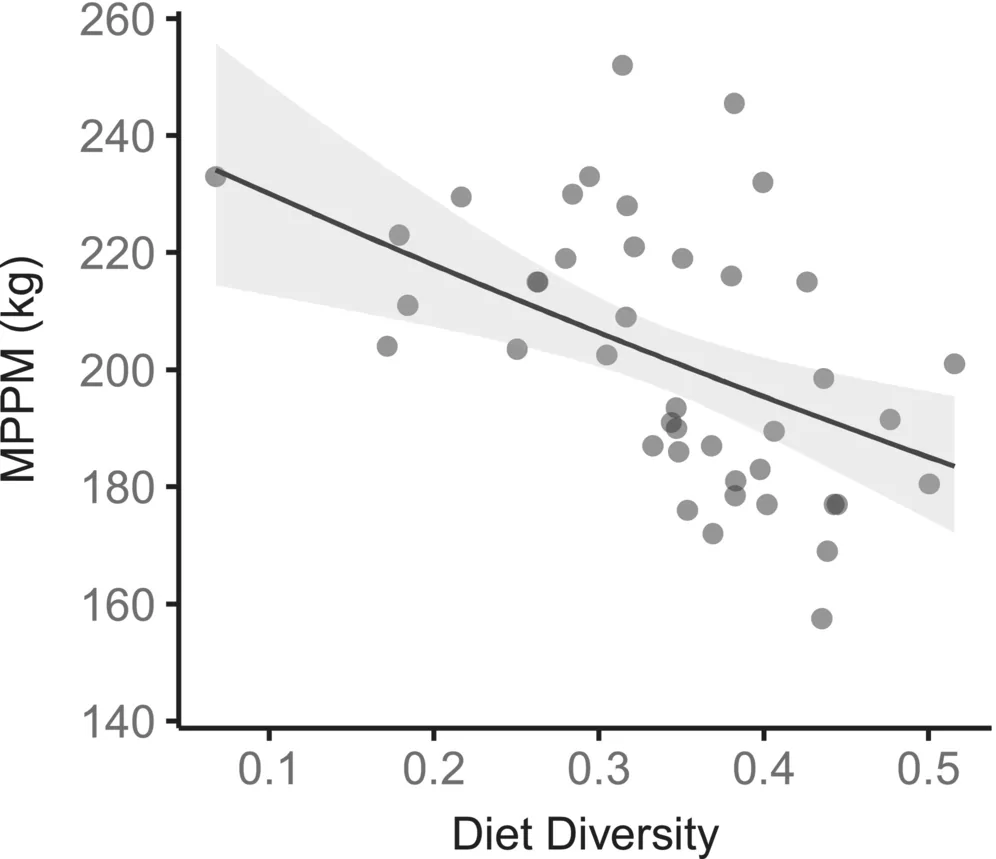
Maternal postpartum mass (MPPM; kg) across QFASA estimated diet diversity from 42 female gray seal ( *Halichoerus grypus* ) deployments. Shaded area represents the 95% CI. Full model results are presented in Table 5.

**TABLE 6:** Summary of the preferred models relating estimated SI ratios to maternal postpartum mass (MPPM; *n* = 40) and pup weaning mass (PWM; *n* = 36) from female gray seal ( *Halichoerus grypus* ) deployments taking place between 1995 and 2018.

Model	δ^13^C		δ^15^N		Mom Age		Pup Stage		Length		MPPM	
	Est.	Std. Error	Est.	Std. Error	Est.	Std. Error	Est.	Std. Error	Est.	Std. Error	Est.	Std. Error
**MPPM ~ δ** ^**13**^ **C + δ** ^**15**^ **N + Length**	0.08*	0.02	−0.03	0.02	—	—	—	—	0.04*	0.02	X	X
MPPM ~ δ^13^C + δ^15^N + MomAge+ Length	0.07*	0.02	−0.04	0.02	0.02	0.03	—	—	0.04*	0.02	X	X
**PWM ~ MomAge + MPPM**	—	—	—	—	−0.07*	0.02	—	—	X	X	0.13*	0.02
PWM ~ MomAge + PupStage + MPPM	—	—	—	—	−0.07*	0.02	0.03	0.03	X	X	0.13*	0.02

*Note:* Coefficient estimates and standard errors for the scaled covariates are provided. Asterisks indicate significance at *p* < 0.05. An “X” indicates that the covariate was not included in the particular model. Models with the highest evidence ratio for each response variable are in bolded font. The model selection tables are presented in Tables 2 and 3.

**FIGURE 3:**
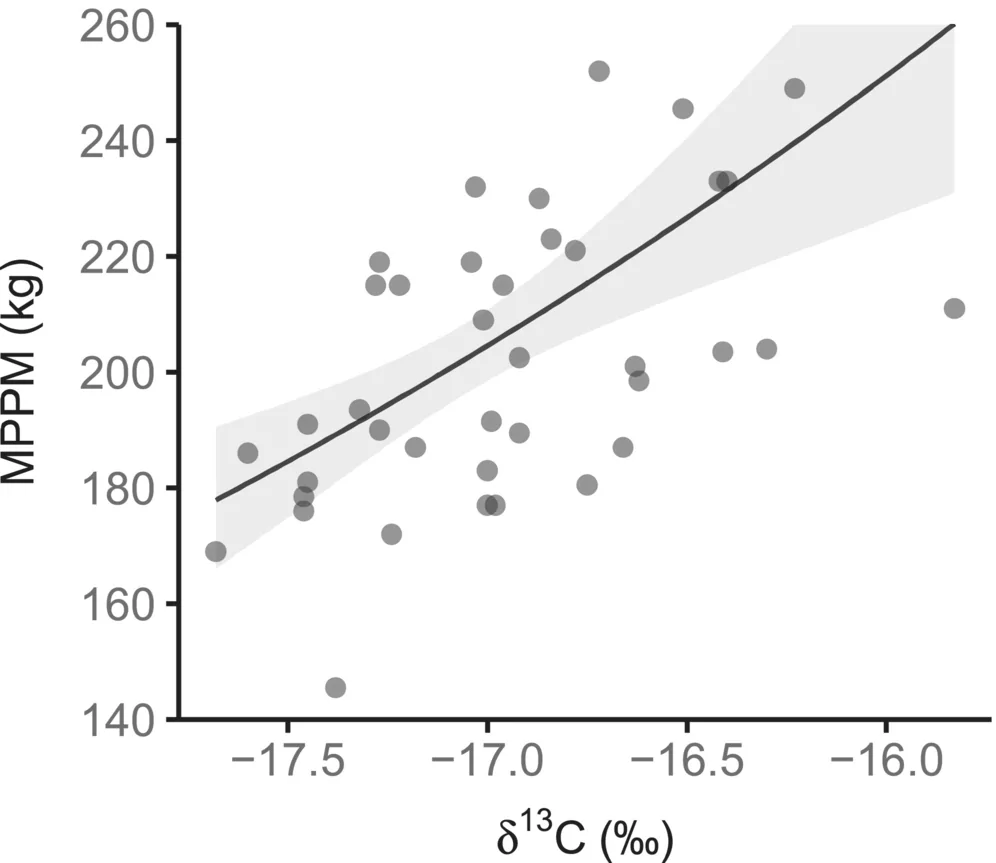
Maternal postpartum mass (MPPM; kg) across estimated δ13C (‰) from 40 female gray seal ( *Halichoerus grypus* ) deployments. Shaded area represents the 95% CI. Full model results are presented in Table 6.

Using female foraging metrics, variation in PWM was best explained by total distance traveled by females along with maternal traits and pup stage (Table 4). However, only MPPM was a significant predictor of variation in PWM. Although diet diversity was retained in the model, only maternal age and MPPM were useful predictors of PWM (Table 5). Neither δ^13^C nor δ^15^N were retained in the preferred model relating SI estimates to PWM (Table 6). Across models using all three sets of covariates, GLMs generally identify maternal age (negative relationship) and MPPM (positive relationship) as the strongest predictors of PWM (Tables 4, 5, 6).

## 4 Discussion

Neither the proportion of time spent in an estimated foraging state, the total distance traveled, nor the number of foraging patches used by gray seal females were useful predictors of MPPM. However, there was indication of a negative relationship between diet diversity and MPPM. Diet diversity increases when food resources become limiting, which is often attributed to among‐individual variation in diet (Schoener 1971; Roughgarden 1972; Krebs et al. 1977; Thompson and Colgan 1990), but there is also evidence of within‐individual diet diversity following the same trend (Svanbäck and Bolnick 2005, 2007). Analysis of the estimated diet of the females involved in this study suggests that prey composition changed little over the study period and that most females primarily consumed redfish or sand lance, with these two species making up approximately 70% of the average diet. While the abundance of these two prey species did show some inter‐annual variation, estimates from summer survey data suggest the abundance of both species appears to have been relatively stable or increasing throughout the study period (Ricard et al. 2022), which may partly explain why dominant prey was not a useful predictor of MPPM. With no evidence of a long‐term decline in preferred prey abundance, we suggest that the variation in diet diversity of the individual gray seal females in this study was due to differences in foraging tactics rather than temporal trends in prey abundance. The lack of relationships among foraging effort, diet characteristics, and PWM suggests that there are multiple ways to achieve reproductive success so long as sufficient energy is stored to support the energy requirements of the pup.

Given that environmental conditions can affect maternal traits, which in turn influence reproductive success, we note that the number of gray seal pups being born on Sable Island was increasing throughout our study period (Hammill et al. 2023). Therefore, our study was conducted during a period of favorable environmental conditions and sufficient resource availability for high reproductive success. Evidence for this comes from the fact that all females involved in this study returned to Sable Island and successfully weaned a pup. Nevertheless, it is possible that environmental variability led to changes in the prey assemblage and prey distribution across the Scotian Shelf between 1995 and 2018 that could have affected foraging effort. However, the high correlation (r_pearson_ = 0.92) between the deployment year and population size during this study did not allow us to separate the effects of year (a proxy for environmental variation) and population size on foraging effort.

We used area‐restricted search behavior to define foraging patches. Although area‐restricted search behavior may reflect foraging effort, it may not correlate well with prey capture success, energy intake or profitability (Allegue et al. 2023; Florko et al. 2023). Considering that QFASA estimates of diet are compositional in nature and do not consider prey‐specific qualities (e.g., prey size), we could not estimate total energy intake over the foraging period, thus preventing us from directly measuring energetic benefits associated with different foraging tactics.

Ideally, we would have used female mass gain over the foraging period leading up to parturition as a measure of female reproductive success. However, females in this colony are known to gain body mass at different rates across seasons (Beck et al. 2003, 2003a, 2003b). Given that females in this study were deployed during the summer and fall for periods ranging from ~3 to 6 months, the mass change observed for different deployments in this study was likely confounded by seasonal and individual differences in energy storage. Furthermore, the diet samples acquired at the time of instrument recovery are believed to only represent the last three to four months of prey consumption, thus mass change during the beginning of summer deployments is likely not reflected in our diet estimates. As such, MPPM was selected as the measure of female reproductive success due to its known importance in reproductive performance (Iverson et al. 1993; Mellish et al. 1999; Pomeroy et al. 1999) and to reduce the effect of the large temporal differences between deployment dates. In the context of MPPM, females lose upwards of 4 kg/day of body mass during lactation (Mellish et al. 1999; Sanchez et al. 2026), making pup age at MPPM measurement an important consideration. Since pup birth dates were not known for many individuals, potentially due to the pup being born before researchers arrived at the island, inclement weather preventing field activities, or the female not being found immediately upon arrival, pup stage at MPPM measurement was included as a covariate in all models.

A negative relationship between diet diversity and MPPM could also arise through individuals receiving a benefit from focusing foraging effort on more profitable prey species. For example, studies on Baltic gray seals have revealed positive relationships between preferred fish prey quality and individual body condition (Kauhala et al. 2017; Kauhala and Kurkilahti 2020). While a mechanism for improved fitness due to reduced diet diversity at the individual level could operate through increased consumption of energy‐rich prey sources, the lack of relationship detected between the energy density of the diet and MPPM suggests that a different mechanism is acting here. Prey size influences the net energy gain associated with specific prey types for a variety of marine predators, including gray seals (Kauhala et al. 2017), harbor seals ( *Phoca vitulina* ; Bowen et al. 2002), harbor porpoises ( *Phocoena phocoena* , Gallagher et al. 2022; Rojano‐Doñate et al. 2024), and Common Murres ( *Uria aalge* , Burke and Montevecchi 2009). However, QFASA estimates of diet generally do not identify differing sizes of particular prey types. Prey profitability also depends heavily on encounter rate and prey capture success (Pyke et al. 1977; Engen and Stenseth 1984). For example, in male Eurasian Kestrels ( *Falco tinnunculus* ), individuals with lower diversity diets had better body condition than males with more generalist diets. This was attributed to restricting the diet to more profitable prey items in terms of prey biomass and capturability (Hochleitner et al. 2025). There is also evidence that Pigeon Guillemots ( *Cepphus columba* ) with more specialized diets have higher fledgling rates than generalist individuals, which the authors suspected was due to increased foraging efficiency (Golet et al. 2000). Litzow and Piatt (2003) found Pigeon Guillemot resting times were higher when foraging on schooling fish rather than non‐schooling fish, allowing flexible time allocation to buffer meal delivery rates against declining food abundance when schooling fish dominated the diet.

Likewise, Little Penguins ( *Eudyptula minor* ) spent more time at foraging patches with lower calorific value prey when these patches were denser and the capture rates were significantly higher than more energy‐rich alternatives (Sutton and Arnould 2022). Lamb et al. (2017) provided evidence that the quantity, particularly through targeting schooling fishes, rather than energy density or lipid content of prey, leads to greater reproductive success in an income‐breeding species, the Brown Pelican ( *Pelecanus occidentalis* ). Similarly, the amount of food received by Rhinoceros Auklet ( *Cerorhinca monocerata* ) chicks was a better predictor of chick growth rates than diet composition (Thayer and Sydeman 2007). In capital breeding pacific gray whales, the probability of individuals being in an apparent foraging state appears to be governed by a trade‐off between prey abundance and quality (Hildebrand et al. 2022), perhaps indicating that foraging strategies targeting abundant or aggregated prey resources are a common and profitable strategy for a wide range of taxa exhibiting different reproductive strategies. Thus, female gray seals who encounter greater numbers of aggregated prey, regardless of prey type, may experience greater mass gain compared to individuals with more diverse diets. However, since our methods of diet estimation do not allow for the estimation of total quantity of prey or energy consumed, these hypotheses remain to be tested.

The positive relationship between δ^13^C and MPPM speaks to the carbon source of the habitat with more enriched carbon signatures generally being associated with benthic species and/or benthic foraging (Sherwood and Rose 2005; Le Loc'h et al. 2008; Kopp et al. 2015). Thus, our results suggest that heavier gray seal females returning to the breeding colony to pup may be associated with more benthic foraging tactics. Tucker et al. (2007) reported similar findings where both adult male and female gray seals appear to shift from more pelagic to benthic diets as body size increases. Although no significant relationship between δ^15^N and MPPM was detected in this study, Tucker et al. (2007) found a positive relationship between δ^15^N, but not δ^13^C, and the estimated rate of fat accumulation during the fall foraging period but no effect of either SI on the percent body fat content of females upon returning to the breeding colony. These results suggest that differing foraging tactics or environments are used by females of different body sizes. Nevertheless, these tactics appear to result in equivalent outcomes in terms of percent body fat at the end of the foraging period. Combining results from this study with those of Tucker et al. (2007), it appears that larger females may have improved access to deeper foraging areas. We speculate that these deeper foraging areas are less well used, potentially reducing intraspecific competition (Breed et al. 2013; Lidgard et al. 2020) leading to improved access to available prey resources. This is also suggested by the negative relationship between diet diversity and MPPM observed in this study and by previous findings that older and larger females may have a competitive advantage in access to more profitable foraging areas (Lidgard et al. 2020). However, δ^13^C values may also vary regionally with other factors, including depth and inshore‐offshore gradients (Kopp et al. 2015; Walters et al. 2021; Espinasse et al. 2022), water temperature and productivity (Walters et al. 2021; Espinasse et al. 2022), and water masses and mesoscale activity (Rodríguez‐Pérez et al. 2023; Gorbatenko and Melnikov 2023).

Although we found evidence that certain aspects of female gray seal foraging tactics are associated with greater MPPM, measures of foraging effort and diet were not useful predictors of PWM. Rather, models consistently identified maternal age and MPPM as the strongest predictors of PWM, as previously reported for gray seals (Bowen et al. 2006). The finding that larger females wean heavier pups has also been observed in the gray seals breeding on North Rona, Scotland (Pomeroy et al. 1999), southern elephant seals (McMahon et al. 2017), and Weddell seals (Wheatley et al. 2006). In otariids and odobenids, several foraging strategies often exist within a species, and individuals often specialize in one or a couple of these strategies (McHuron et al. 2016; Jeanniard‐du‐Dot and Guinet 2021). While specialization in one strategy may provide fitness benefits when resource availability is sufficient, as seen with lower diet diversity being associated with higher MPPM in this study, the existence of multiple foraging strategies within a species may be an evolutionary buffer against changing environmental conditions (Jeanniard‐du‐Dot and Guinet 2021). As such, data collected during periods of poor environmental conditions or resource availability may lead to the emergence of other foraging tactics.

## Author Contributions

**M. Henry‐Adams:** formal analysis (lead), investigation (equal), writing – original draft (lead), writing – review and editing (equal). **W. D. Bowen:** conceptualization (lead), formal analysis (equal), funding acquisition (lead), investigation (equal), methodology (lead), writing – original draft (lead), writing – review and editing (equal). **C. E. den Heyer:** data curation (lead), formal analysis (equal), funding acquisition (supporting), investigation (equal), methodology (equal), writing – review and editing (equal). **S. L. C. Lang:** data curation (equal), formal analysis (equal), investigation (equal), methodology (equal), writing – review and editing (equal). **S. J. Iverson:** funding acquisition (supporting), investigation (equal), methodology (equal), writing – review and editing (equal).

## Funding

This work was supported by a Major Sciences Initiative grant from Canada Foundation for Innovation to the Ocean Tracking Network, Discovery Grants to W.D.B. and S.J.I. from the Natural Sciences and Engineering Research Council of Canada, and by the Department of Fisheries and Oceans, Canada.

## Ethics Statement

This research was conducted in accordance with the Canadian Council on Animal Care (CCAC) guidelines on the care and use of marine mammals in research. The research protocols for these studies were approved by Dalhousie University's Committee on Laboratory Animals and by the Department of Fisheries and Oceans, Canada Animal Use Committee.

## Conflicts of Interest

The authors declare no conflicts of interest.

## Supporting information


**Data S1:** ece374536‐sup‐0001‐supinfo.xlsx.

**Appendix A** Summary of gray seal diet estimation methods and the cluster analysis used to identify diet groups.
**Figure A1** Overall diet contribution, expressed as an average % of total diet composition, by prey species summed across 46 female gray seal deployments between 1995 and 2018.
**Figure A2** Plot displaying the results of the silhouette analysis (a) and elbow plot (b) performed to identify the optimal number of clusters for analysis of diet groups.
**Figure A3** Average estimated proportion of the diet comprised of each prey species making up ≥ 5% of the diet in either cluster.
**Figure A4** Scatterplot displaying the distribution of estimated diet diversity and energy density from 42 female gray seal diets included in this study.
**Figure A5** Scatterplot displaying the distribution of estimated δ^13^C and δ^15^N values from 40 female gray seals included in this study.

## Data Availability

The data that supports the findings of this study are available in the Supporting Information.
