# Supplementary material for: Influence of Foraging Effort and Diet on Maternal Energy Stores and Offspring Weaning Mass in Gray Seals (Halichoerus grypus)
Source: Ecol Evol. 2026 Oct 28;16(11):e74536. doi: 10.1002/ece3.74536 (PMC13715072; doi:10.1002/ece3.74536)
Supplement: Supplementary file 1 [file ECE3-16-e74536-s002.docx]

***Appendix A – Summary of grey seal diet estimation methods and the cluster analysis used to identify diet groups.***

Here we describe the estimated diets of 46 adult female grey seals estimated using quantitative fatty acid signature analysis (QFASA) from blubber samples obtained at three or more days postpartum. The methods used to estimate diet using QFASA are presented in the methods section of Henery-Adams et al. (submitted). The estimated contribution of individual prey species towards all combined diets is summarized in Figure A1, where all species composing ~5% of the average diet are shown with all other prey species making up a smaller proportion of the average diet grouped into the “other” category. Across all individuals, redfish, northern sand lance, Atlantic cod, pollock and capelin were found to be the most notable prey species contributing to the average estimated diet.

To determine whether the estimated diets of individuals could be grouped based on compositional similarities, a k-means cluster analysis was performed without a pre-determined number of clusters. Since QFASA diet estimates are compositional in nature, a centered log-ratio transformation was applied to the data before cluster analysis. The optimal number of clusters, as determined by both an elbow plot method and silhouette analysis (Fig. A2), revealed two distinct diet groups. A k-means cluster analysis with k=2 clusters was then performed. All species identified as composing ≥ 5% of the diet, on average, of either diet group were then used to describe each group. A summary of prey species defining each identified cluster are presented in Figure A3. Based on these summary statistics (Fig. A3), each diet cluster was classified based on the prey species comprising the largest average proportion and the most prevalent prey species within the estimated diet of each individual in each cluster. This resulted in diets within cluster 1 being classified as redfish dominant diets and diets within cluster 2 being classified as sand lance dominant diets.

We then calculated the energy density and diversity of each female’s diet using the fall proximate composition of prey species (Beck et al. 2007). The energy density (kJ/g) of prey tissue was estimated using the equation:

*Equation 1)*:

$$energy density=\left( \left( \frac{\%lipid}{100} \right)\cdot39.3 kJ/g \right)+\left( \left( \frac{\%protein}{100} \right)\cdot23.6 kJ/g \right)$$

Following the methods presented in Beck et al. (2007), diet diversity was calculated using a standardized Shannon–Weiner Index (Krebs 1989, Newton-Fisher 1999):

*Equation 2)*:

$$H^{'}=\left( -\sum_{s}^{j} p_{j} ln p_{j} \right)/{ln S}$$

where *H*′ is the standardized measure of diversity, *p*_j_ is the proportion of prey species *j* in the diet and *S* is the total number of prey species consumed by all individuals. The distribution of energy density and diet diversity values of each diet used in this study are presented in Fig. A4. Note that while 46 female diets were available for the cluster analysis presented in Henry-Adams et al. (submitted) and summarized above, Figures A4 and A5 represent the data (n = 42) used in this study, which excludes four of the females included in the dataset for Henry-Adams et al. (submitted) due to the need for recorded reproductive characteristics for each female in this study.

We also collected skin samples from 40 of the deployments for stable isotope (SI) analysis at instrument recovery. SI analysis was performed as described in Tucker et al. (2007). Stable carbon and nitrogen ratios are expressed in delta (δ) notation, expressed as the parts per thousand (‰) deviation from a standard material. δ^13^C values were corrected for known fractionation rates for seal skin tissue. The observed values δ^13^C and δ^15^N from all 40 individuals included in this study are presented in Fig. A5.

Figure A1	Overall diet contribution, expressed as an average % of total diet composition, by prey species summed across 46 female grey seal deployments between 1995 to 2018.

Figure A2	Plot displaying the results of the silhouette analysis (a) and elbow plot (b) performed to identify the optimal number of clusters for analysis of diet groups.

Figure A3	Average estimated proportion of the diet comprised of each prey species making up ≥5% of the diet in either cluster.

Figure A4	Scatterplot displaying the distribution of estimated diet diversity and energy density from 42 female grey seal diets included in this study.

Figure A5	Scatterplot displaying the distribution of estimated δ^13^C and δ^15^N values from 40 female grey seals included in this study.
